# Human Serum Albumin Nanoparticles as 3,6-Diazaphenothiazine Delivery System: Preparation and Interaction Studies

**DOI:** 10.3390/molecules31142541

**Published:** 2026-07-22

**Authors:** Karolina Kulig, Aleksandra Owczarzy, Patrycja Sarkowicz, Patrycja Piśla, Katarzyna Piordas, Emilia Martula, Małgorzata Jeleń, Beata Morak-Młodawska, Magdalena Ziąbka, Wojciech Rogóż, Małgorzata Maciążek-Jurczyk

**Affiliations:** 1 Department of Physical Pharmacy, Faculty of Pharmaceutical Sciences in Sosnowiec, Medical University of Silesia in Katowice, 40-055 Katowice, Poland; kkulig@sum.edu.pl (K.K.); aowczarzy@sum.edu.pl (A.O.); s83464@365.sum.edu.pl (P.S.); s83448@365.sum.edu.pl (P.P.); s83447@365.sum.edu.pl (K.P.); wrogoz@sum.edu.pl (W.R.); 2 Department of Organic Chemistry, Faculty of Pharmaceutical Sciences in Sosnowiec, Medical University of Silesia in Katowice, 40-055 Katowice, Poland; emilia.martula2903@gmail.com (E.M.); manowak@sum.edu.pl (M.J.); bmlodawska@sum.edu.pl (B.M.-M.); 3 Department of Condensed Matter Physics, Faculty of Physics and Applied Computer Science, AGH University of Krakow, 30-059 Krakow, Poland; ziabka@agh.edu.pl

**Keywords:** albumin, nanoparticles, spectroscopy, microscopy, calorimetry, 10*H*-3,6-diazaphenothiazine

## Abstract

Plasma proteins are becoming more and more popular among researchers due to their minimal toxicity and immunogenicity. The largest percentage of plasma proteins is human serum albumin (HSA). HSA is widely used as a drug carrier due to its biocompatibility and specific affinity to cancer cells. 10*H*-3,6-diazaphenothiazine (DAPT) is a newly synthesized phenothiazine derivative with promising anticancer activity. The main aim of this study was to encapsulate the DAPT into human serum albumin nanoparticles (DAPT-HSA-NPs) as well as to study DAPT interaction with HSA based on spectroscopic, microscopic, and calorimetric techniques. HSA nanoparticles with DAPT (DAPT-HSA-NPs) were prepared using the desolvation method, and this reaction was accompanied by a thermal transition. High encapsulation efficiency of DAPT into the HSA-NPs (DAPT-HSA-NPs) was obtained (~100%) and its release kinetics from the DAPT-HSA-NP system followed the zero-order kinetic model. Both nanoparticle preparation (HSA-NPs) and HSA interaction with DAPT (DAPT-HSA) resulted in changes in the HSA secondary structure. Moreover, the process of DAPT binding to HSA was exothermic (ΔH [kcal·mol^−1^] < 0), and DAPT probably formed a static complex with HSA (kq [L·mol^−1^·s^−1^] > 10^12^) with moderate affinity (K_a_ [L·mol^−1^] of the order of 10^4^). Despite reports on human serum albumin nanoparticles (HSA-NPs) and 10*H*-3,6-diazaphenothiazine (DAPT), no studies on DAPT encapsulation into HSA-NPs have been published. Therefore, HSA-NPs as a 3,6-diazaphenothiazine delivery system, including preparation methods and interaction analysis, have been evaluated.

## 1. Introduction

Human serum albumin (HSA) represents the largest fraction of plasma proteins, accounting for approximately 60% of total plasma protein content, and has s half-life of 19 days [1]. HSA is a non-glycosylated globular protein of 585 amino acids with a molecular weight of 66.5 kDa and an asymmetric heart-shape structure [2]. It is organized in three homologous domains (I–III), each divided into subdomains A and B and two specific binding sites (Sudlow sites I and II). Sudlow site I is found in subdomain IIA, whereas site II is located in subdomain IIIA [1,2,3]. Despite its molecular weight and the quaternary structure, HSA can extravasate from the bloodstream, allowing it to enter the lymphatic system and accumulate in sites of inflammation or within the tumor microenvironment [2].

Due to its ability to transport endogenous and exogenous ligands, as well as its high solubility, stability, low immunogenicity, and long half-life, HSA is a suitable candidate for the development of drug delivery systems [1]. Moreover, as a biomaterial, it provides protective functions for hydrophobic drugs [4]. The globular structure of HSA enables the facile formulation of nanoparticles (HSA-NPs) by the desolvation method. These nanoparticles are biodegradable due to the inherent properties of HSA and remain soluble despite the cross-linking process [4].

**Figure 1:**
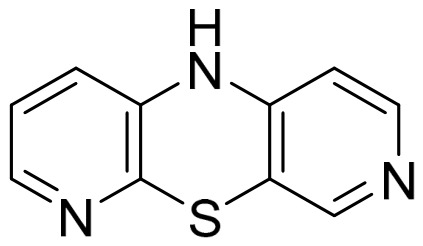
Structure of 10*H*-3,6-diazaphenothiazine (DAPT).

Phenothiazines are widely known antipsychotic drugs that revolutionized psychiatry in the last century, allowing for the pharmacological treatment of schizophrenia and manic states [5]. They also exhibit valuable antihistamine activity. Despite the passage of many years, this group of drugs is still used in medicine. Phenothiazines are also popular because they exhibit other valuable biological properties, such as antibacterial, antiviral, anticonvulsant, or anti-inflammatory properties [6,7]. 10*H*-3,6-diazaphenothiazine (Figure 1) is a phenothiazine derivative that demonstrated promising in vitro anticancer activity in cytotoxicity studies against glioblastoma SNB-19, melanoma C-32, and breast cancer MCF-7 cell lines, with IC_50_ values of 0.46 and 0.72 μg·mL^−1^, and relatively low toxicity against the normal fibroblast cell line HFF-1. Gene expression analysis (*H3*, *TP53*, *CDKN1A*, *BCL-2*, and *BAX*) performed by RT-qPCR confirmed the antiproliferative activity of this compound, indicating activation of the p53 pathway in cancer cells, inducing cell cycle arrest [8]. Lipophilicity and other pharmacokinetic parameters of the test compound were determined both in silico and experimentally using reversed-phase thin-layer chromatography (RP-TLC). The experimentally determined lipophilicity parameter, expressed as logP_TLC_, was 0.952 at physiological pH (7.4). This value indicates relatively low lipophilicity of the compound, suggesting a balanced distribution between aqueous and lipid environments [9].

Scientists are constantly searching for new drug delivery methods. Albumin has emerged as one of the most promising candidates due to the development of novel drug delivery strategies [10]. To date, attempts have been made to develop albumin-based drug delivery systems, such as albumin–drug conjugates, albumin-binding prodrugs, albumin fusion proteins, and albumin-based nanoparticles [10]. The encapsulation of new derivatives of established drugs is also a new trend in research. COP-22 curcumine derivative might be an example of encapsulation of newly synthesized derivative into albumin NPs [11] or encapsulation of 7-ethyl-10-hydroxyl camptothecin into conjugate/human serum albumin nanoparticles [12]. In the field of nanoparticle research, in addition to basic physicochemical characterization, researchers often perform supplementary analyses to gain a deeper understanding of the mechanisms underlying these drug delivery systems. Analysis of nanoparticles can provide valuable insights into biological processes and mechanisms that occur in vivo. Nanomaterials such as metal nanoparticles exhibit strong absorption in the visible wavelength range but are generally achiral and therefore lack chiroptical properties. In contrast, biopolymer nanoparticles, including protein-based nanoparticles, are inherently chiral, allowing changes in their secondary structure during nanoparticle preparation to be monitored using chiroptical techniques [13,14]. Circular dichroism (CD) spectroscopy is an excellent tool to study proteins’ structure in solutions; however, it is also used to examine structural changes in albumin after the albumin nanoparticle preparation process [13,15,16].

The aim of this project was to propose HSA nanoparticles as a form of DAPT delivery system (DAPT-HSA-NPs) and to conduct in vitro spectroscopic and calorimetric analysis of both the ligand release and the nature of its interaction with the main transport protein. The experiment is basic in its character, while from a scientific point of view, it is novel due to the fact that no studies on DAPT encapsulation into HSA-NPs have been published and it encourages further research. This study is a continuation of our research on delivery systems for new phenothiazine derivatives; therefore, the methodology follows that of our previous work. This publication also provides further information on the interactions between HSA and DAPT.

## 2. Results

### 2.1. Nanoparticle Preparation and Characterization

**Figure 2:**
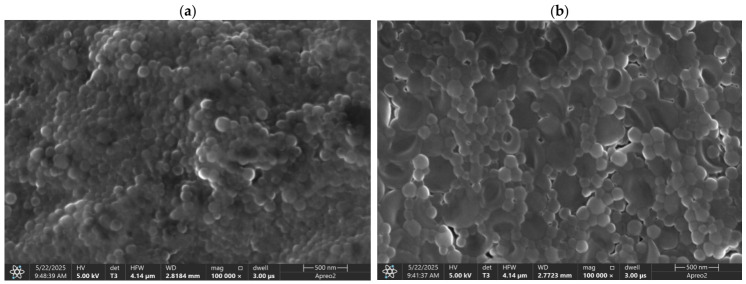
Size and morphology of HSA-NPs (**a**) and DAPT-HSA-NPs (**b**).

HSA-NPs and DAPT-HSA-NPs were prepared according to the mentioned procedure. The encapsulation efficiency of the DAPT into the nanoparticles was 98.58 ± 0.36% (*n* = 3; average ± SD). Particle diameter was measured as 114 ± 24.91 nm for HSA-NPs and 120 ± 26.31 nm for DAPT-HSA-NPs. The morphology of nanoparticles was described as smooth and spherical without any pores on their surfaces. Particle size and morphology are shown in Figure 2a,b.

### 2.2. In Vitro Drug Release

**Figure 3:**
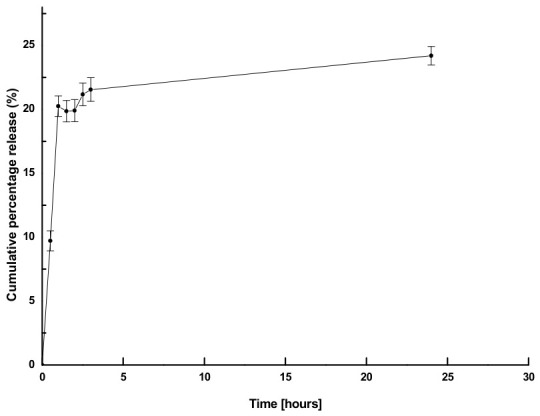
DAPT release from HSA nanoparticles.

Release of DAPT from DAPT-HSA-NPs was analyzed with the use of UV-Vis spectroscopy. To mimic physiological conditions, solutions of DAPT-HSA-NPs were introduced into the releasing medium and gently stirred at 310 K. The results have been shown in Figure 3.

According to the SwissADME in silico analysis, lipophilicity (consensus LogP_o/w_) was 1.92. The predicted aqueous solubility values were as follows: ESOL model: LogS = −3.03, corresponding to 0.187 mg·mL^−1^ (9.28 · 10^−4^ mol·L^−1^); Ali model: LogS = −3.03, corresponding to 0.186 mg·mL^−1^ (9.23 · 10^−4^ mol·L^−1^); and SILICOS-IT model: LogS = −4.33, corresponding to 0.0094 mg·mL^−1^ (4.68 · 10^−5^ mol·L^−1^). These predictions indicate that DAPT may exhibit limited-to-moderate aqueous solubility, depending on the applied model.

**Figure 4:**
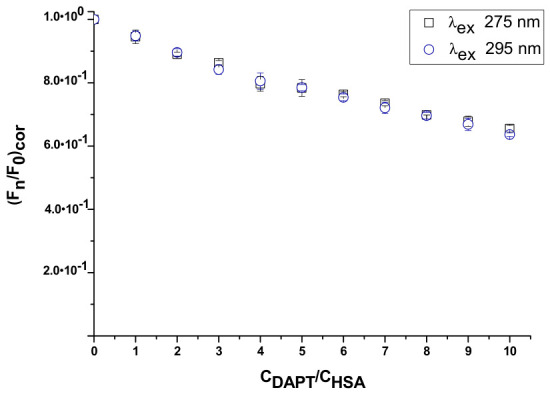
Fluorescence quenching curves of HSA at molar concentration 2 · 10^−6^ mol·L^−1^ in the presence of DAPT at increasing concentration from 0 mol·L^−1^ to 2 · 10^−5^ mol·L^−1^ (λ_ex_ 275 nm and λ_ex_ 295 nm).

The DAPT release from DAPT-HSA-NPs occurred within 0.5 h of the start of the experiment, and the last measurement point was collected at the 24th hour of the experiment. The cumulative release rate for 24 h was 24.19% (Figure 4). Different release kinetics models were fitted for the release profile. Based on the regression coefficient (R^2^) and rate constant values (K), the best kinetics model was assigned. The results of the mathematical analysis are shown in Table 1.

### 2.3. Fluorescence Spectra Measurements

The steady-state fluorescence spectra (Figure S1) of the DAPT-HSA system were recorded in order to determine the interaction between 10*H*-3,6-diazaphenothiazine (DAPT) and human serum albumin (HSA). Based on it, the HSA fluorescence quenching curves in the presence of DAPT at increasing concentration are plotted and presented on Figure 4.

Based on the fluorescence quenching curves, it can be observed that the increasing concentration of DAPT decreased the HSA fluorescence intensity. In addition, it was observed that the fluorescence quenching curves overlap at both excitation wavelengths (λ_ex_ 275 nm and λ_ex_ 295 nm) in the entire range of DAPT concentrations. Subsequently, based on the recorded steady-state fluorescence spectra (Figure S1), the percentage of fluorescence quenching of HSA at the highest concentration of DAPT was calculated and is presented in Table 2.

Based on the data collected in Table 2, it can be observed that DAPT quenched the HSA fluorescence at about 36%, at both λ_ex_ 275 nm and λ_ex_ 295 nm excitation wavelengths. In addition, based on the steady-state fluorescence spectra as a result of the presence of DAPT, a shift in the short- (blue shift) and long-wavelength (red shift) spectra was observed in the fluorescence intensity maxima of HSA at λ_ex_ 275 nm and λ_ex_ 295 nm, respectively. Short-/long-wavelength spectral shifts indicated changes in the amino acid environment. In order to confirm potential alterations due to the external factors, the spectral parameter A (Equation (8)) was calculated and is presented in Table 3.

**Figure 5:**
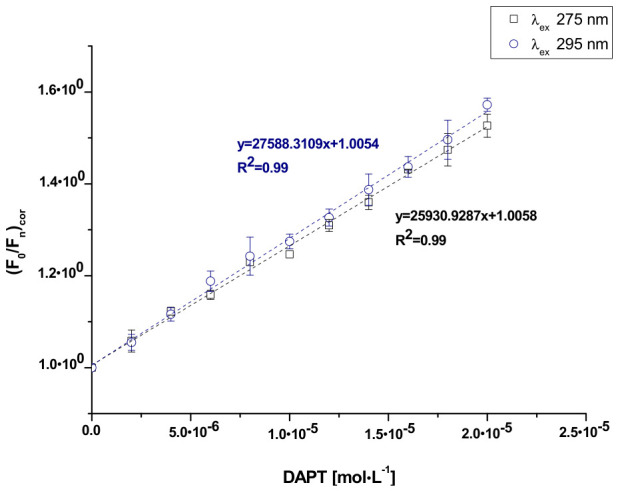
Stern–Volmer curves of HSA at 2 · 10^−6^ mol·L^−1^ concentration in the presence of DAPT at increasing concentration from 0 mol·L^−1^ to 2 · 10^−5^ mol·L^−1^ (λ_ex_ 275 nm and λ_ex_ 295 nm).

Based on the data collected in Table 3, it was observed that a decrease in the fluorescence intensity maxima of HSA was correlated with a decrease in the value of the spectral parameter A at the excitation wavelength λ_ex_ 275 nm, and vice versa (λ_ex_ 295 nm). In order to determine the type of fluorescence quenching effect (static and/or dynamic) for the DAPT-HSA system, the Stern–Volmer equation was used (Equation (9)). The obtained curves are presented in Figure 5.

The Stern–Volmer plots of the DAPT-HSA system at both λ_ex_ 275 nm and λ_ex_ 295 nm excitation wavelengths had a rectilinear course, and it was challenging to estimate the nature of the fluorescence quenching. Accordingly, using the Stern–Volmer equation (Equation (9)), the Stern–Volmer constant (K_S-V_ [L·mol^−1^]) and the bimolecular quenching rate constant (k_q_ [L·mol^−1^·s^−1^]) at both excitation wavelengths λ_ex_ 275 nm and λ_ex_ 295 nm were calculated and are summarized in Table 4.

**Figure 6:**
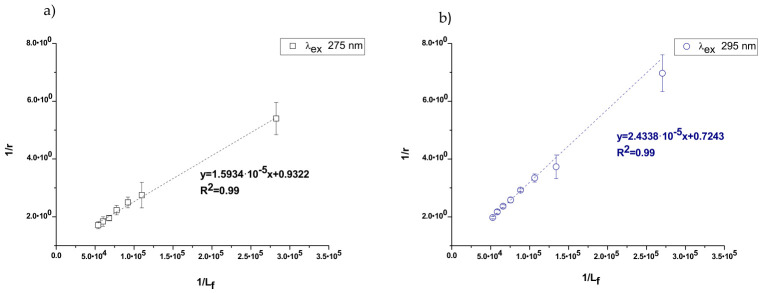
The Klotz plots for the DAPT-HSA system (λ_ex_ 275 nm (**a**) and λ_ex_ 295 nm (**b**)).

From the data collected in Table 4, it can be observed that the K_S-V_ and k_q_ values of the DAPT-HSA system are of the order of magnitude 10^4^ and 10^12^, respectively, at both excitation wavelengths λ_ex_ 275 nm and λ_ex_ 295 nm. To determine the binding parameters for the DAPT-HSA system, based on Equation (10), the Klotz curves have been plotted and are presented in Figure 6. On this basis, the association constant (K_a_ [L·mol^−1^]) and the number of binding site classes (n) were calculated and are shown in Table 5.

Data collected in Table 5 indicate the same order of magnitude of the association constant (10^4^ [L·mol^−1^]), and the number of binding site classes (n) oscillated around 1, at both excitation wavelengths λ_ex_ 275 nm and λ_ex_ 295 nm.

### 2.4. NanoITC Measurements

**Figure 7:**
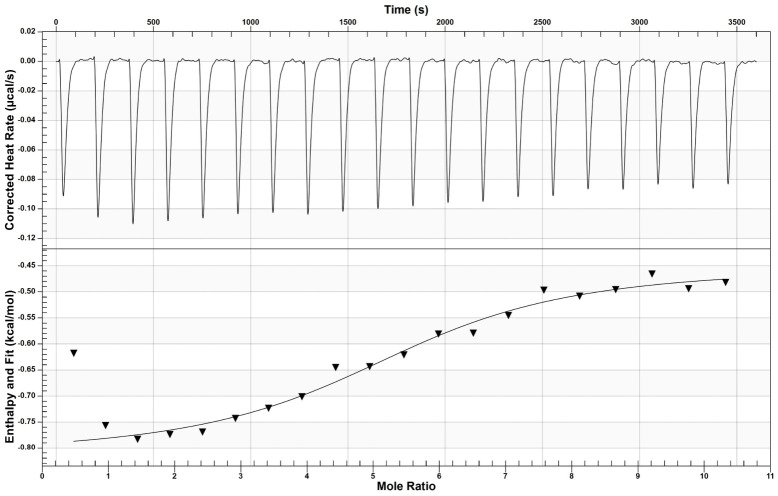
NanoITC thermogram of HSA in the presence of DAPT. The upper figure presents the corrected raw heat data obtained from the consecutive injections, and the lower figure presents the binding isotherm created by plotting areas of the heat peak in relation to the molar ratio DAPT:HSA. The lines present the best fit of the models used.

The character of the interaction in the DAPT-HSA complex was determined using nanocalorimetric measurements. Thermograms of HSA in the presence of DAPT are shown in Figure 7.

Calorimetric parameters accompanying the complex formation reaction were K_a_ = (8.72 ± 0.37) · 10^4^ L·mol^−1^, n = 5.10 ± 1.23, ΔH = −0.45 ± 0.15 kcal·mol^−1^, ΔS = 21.11 ± 0.58 cal·mol^−1^·K^−1^, ΔG = −6.74 ± 0.02 kcal·mol^−1^.

### 2.5. NanoDSC Measurements

**Figure 8:**
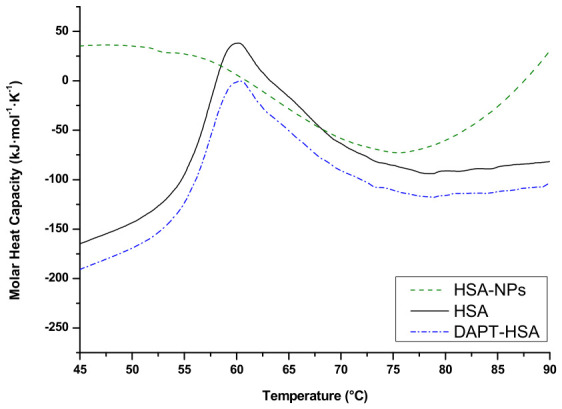
Differential scanning calorimetric thermograms of HSA-NPs, HSA, and DAPT-HSA.

Each thermogram showed one denaturation peak for each sample tested (Figure 8). The denaturation temperatures $T_{D}$ and enthalpy change ΔH of different denaturation peaks were 60.95 ± 0.11 °C, 421 ± 2.69 kJ·mol^−1^ and 1711 ± 407.29 kJ·mol^−1^, 65.65 ± 6.21 °C for DAPT-HSA and HSA-NPs, respectively. The denaturation temperature $T_{D}$ and enthalpy change ΔH of HSA peaks were 60.93 °C and 404 kJ·mol^−1^ (due to low SD values, it was omitted).

### 2.6. Circular Dichroism Measurements

**Figure 9:**
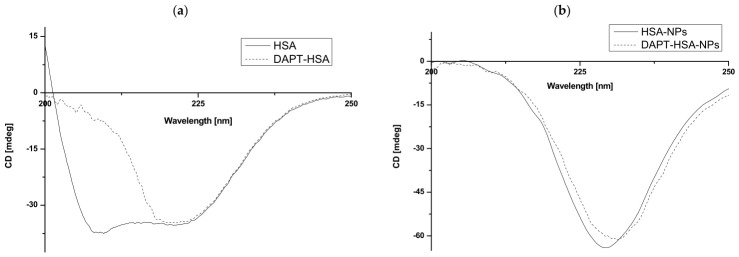
CD spectra of HSA and DAPT-HSA (**a**) and HSA-NPs and DAPT-HSA-NPs (**b**).

Circular dichroism (CD) spectroscopy was used to determine changes in the secondary structure of human serum albumin (HSA), human serum albumin in the presence of DAPT (DAPT-HSA), human serum albumin nanoparticles (HSA-NPs), and human serum albumin nanoparticles with encapsulated DAPT (DAPT-HSA-NPs). The results are shown in Figure 9 and Table 6.

As can be seen, the ΘMRW value increased under the influence of HSA binding to DAPT and after nanoparticle synthesis. DAPT encapsulation further increased the ΘMRW value regardless of wavelengths.

## 3. Discussion

Human serum albumin (HSA) is a negatively charged globular protein with a circulation half-life of 19 days and high solubility [1]. Because of its ability to transport endogenous and exogenous ligands and other advantages mentioned previously, it has been largely applied as a system for drug delivery, both in native (HSA) and modified (HSA nanoparticles, HSA-NPs) forms [2]. The most popular method of HSA nanoparticle preparation is the desolvation method. This method is not time-consuming and does not require the use of surfactants. Moreover, it is reproducible and relatively simple, and has a wide application in the encapsulation of hydrophilic and hydrophobic drugs [4]. On the other hand, desolvation requires the use of organic solvents (most commonly methanol, ethanol, or acetone), which induce the precipitation of albumin and are a key component of the process. These solvents must be thoroughly removed from the final preparation. Despite the partial evaporation of volatile solvents during incubation lasting several hours, residual organic solvents may affect the safety of the product and make it difficult to meet regulatory requirements [17,18]. Nanoparticles formed immediately after desolvation are unstable. Therefore, they usually require the use of cross-linking agents, such as glutaraldehyde, which raises concerns due to its potential cytotoxicity and the need to remove it completely from the final product [17,19,20]. It should be noted, however, that the amount of glutaraldehyde used is unlikely to have a negative impact on either the patient’s body or the technologist working on the production of glutaraldehyde-crosslinked NPs.

Albumin-based nanoparticles (HSA-NPs) are characterized by a high ligand encapsulation efficiency (EE) [4]. In our previous studies, the EE of 10*H*-2,7-diazaphenothiazine into BSA nanoparticles was 66.67% ± 6.11 [21], while that of 10-(2′-pyrimidyl)-3,6-diazaphenothiazine into HSA nanoparticles was 99.65 ± 0.05% [22]. Patel et al. [23] encapsulated benzothiazinone (IR 20, IF 274, FG 2, and AR 112) into HSA nanocarriers. They used two methods of encapsulation: the modified desolvation method and the modified desolvation method with NaOH added to the albumin solution. Using the first modification of the desolvation method, the EE was within the range of 9–55%, while for the second modification of the desolvation method with NaOH, the EE was within the scope of 37–60%. Phenothiazine derivative encapsulation efficiency depends on the substituents contained in the ligand structure. Because the 10*H*-3,6-diazaphenothiazine (DAPT) encapsulation efficiency was much higher (98.58 ± 0.36%) than that published in the literature, the differences may result from the different structure of the encapsulated compounds and minor modifications in the method of obtaining nanoparticles.

The morphology of the nanoparticles was studied using scanning electron microscopy (SEM), and as it is presented in Figure 2a,b that the nanoparticle size for DAPT-HSA-NPs was 120 ± 26.31 nm, while without ligand, the NP size (HSA-NPs) was 114 ± 24.91 nm. Based on the comparison of nanoparticle size to our previous studies, it can be concluded that regardless of whether the ligand is encapsulated or not, the nanoparticles diameter is about 100 nm and larger [21]. Albumin nanoparticles are spherical in shape and have a smooth surface. This has also been confirmed in other studies [24,25,26,27]. The properties of the resulting nanoparticles depend on a number of parameters of the synthesis process. It is possible that, although the desolvation method favors the production of nanoparticles only slightly larger than 100 nm, variations in the parameters may lead to deviations from the previously designed parameters. It is therefore important that the procedure be repeated in other, independent studies [28].

When examining the ligand release from DAPT-HSA-NPs at pH 7.4 (Figure 3), a similar trend (after 24 h with a lower value—24.19%) was observed as in our previous studies when the release of 2,7-DAPT (54.77%) was analyzed. However, models of the release mechanism of 2,7-DAPT at any of the pHs presented [21] are not comparable to those presented in this paper. The relatively low level of DAPT release may result from a combination of factors, including interactions between DAPT and the HSA nanoparticle matrix. The lipophilicity of DAPT was evaluated using both experimental and computational approaches. The experimentally determined lipophilicity parameter obtained by reversed-phase thin-layer chromatography (RP-TLC) at physiological conditions was logP_TLC_ = 0.952, indicating moderate lipophilic properties. In agreement with this result, SwissADME analysis predicted a consensus LogP_o/w_ value of 1.92, also suggesting moderate lipophilicity. The difference between these values may be attributed to methodological differences between the experimental RP-TLC approach and computational estimation of the octanol/water partition coefficient. The possibility of DAPT adsorption onto the membrane filter, during the release process using the sampling and separation method, cannot be completely excluded; however, considering the moderate lipophilicity of DAPT and its predicted limited-to-moderate aqueous solubility, interactions with the membrane material may occur only to a limited extent. The predicted solubility values ranged from 0.0094 mg·mL^−1^ (SILICOS-IT model) to 0.187 mg·mL^−1^ (ESOL model), suggesting limited-to-moderate aqueous solubility depending on the applied prediction model. Based on these values and the amount of DAPT incorporated into the nanoparticles, the concentration of released DAPT during the release experiment was below the predicted solubility range, suggesting that sink conditions were maintained. Therefore, the limited cumulative release is unlikely to be attributed solely to insufficient solubility of free DAPT in the release medium. Instead, the observed profile may be related to the retention of DAPT within the HSA nanoparticle structure, including hydrophobic interactions and possible binding to albumin domains, which can reduce the diffusion rate of the drug molecules into the surrounding medium.

Owing to the Korsmeyer–Peppas model for noncylindrical geometry and nonthin film layers of matrices, to estimate the mechanism of DAPT release from nanocarriers, the values of the diffusional exponent n were found [29]. Based on the Korsmeyer–Peppas model (Table 1), similarly to 5-fluorouracil DAPT, the release process from DAPT-HSA-NPs can be described as Super Case II transport (0.85 < n < 1), indicating that polymer relaxation, matrix restructuring, and solvent penetration processes contribute significantly to drug release. The very high R^2^ value for the Hixson–Crowell model indicates that the release is primarily associated with the erosion or dissolution of the albumin nanoparticles, leading to a reduction in their surface area. However, the slightly lower R^2^ value obtained for the Higuchi model suggests that the release process is also associated with the diffusion of 3,6-DAPT through the nanoparticle matrix [13,30]. Gohar et al. [31] obtained a similar model fit for the release of latanoprost from polymeric micelles. Similarly, studies investigating 5-fluorouracil release from chitosan nanoparticles demonstrated a release profile consistent with the Higuchi model, indicating drug diffusion through the nanoparticle matrix as an important mechanism [32]. The estimated lipophilicity of DAPT is considerably below the upper limit defined by Lipinski’s Rule of Five (logP ≤ 5), which is one of the key criteria used to assess the drug-likeness and oral bioavailability of potential therapeutic agents. Compounds with excessive lipophilicity often exhibit poor aqueous solubility, increased plasma protein binding, rapid metabolic clearance, and a greater risk of nonspecific interactions with biological targets. In contrast, the moderate lipophilicity estimated for the investigated compound may contribute to favorable absorption, distribution, and pharmacokinetic behavior. The relatively low lipophilicity may also reduce the likelihood of excessive accumulation in fatty tissues and minimize the risk of undesirable off-target effects associated with highly lipophilic molecules. Despite the moderate predicted LogP_o/w_ value, the aromatic heterocyclic structure of DAPT may promote interactions with albumin and contribute to its retention within the nanoparticle matrix. Taken together, these findings suggest that the limited release of DAPT from HSA nanoparticles results from a balance between matrix–drug interactions, diffusion processes, and the intrinsic solubility characteristics of the compound.

The fluorescence technique enables the study of interaction between the ligand and carrier proteins in the primary high-affinity binding site. In the specified measurement conditions, the proteins were characterized by the wavelength of maximum fluorescence intensity, the intensity of fluorescence emission spectra in the wavelength scale, and the spectra shape [33]. Changes in fluorescence intensity provide significant information regarding the fluorophores present in macromolecules [34]. In the current study, the fluorescence quenching of HSA was observed as a result of the increasing concentrations of DAPT at both excitation wavelengths (Figure S1). Based on the HSA steady-state fluorescence spectra in the presence of DAPT at increasing concentration, the fluorescence quenching curves have been plotted (Figure 4). The obtained curves are characterized by the same course; in addition, the calculated percentage of HSA fluorescence quenching in the presence of DAPT is also comparable for both excitation wavelengths (Table 2). According to the literature, the excitation wavelength λ_ex_ 275 nm induces fluorescence of both tyrosyl residues and one tryptophanyl residue (Trp-214) present in HSA molecules, whereas the excitation wavelength λ_ex_ 295 nm exclusively induces fluorescence Trp-214. A similar course of fluorescence quenching curves and comparable percentage of HSA quenching at both excitation wavelengths in the presence of the ligand, as in the present work, has also been observed by Owczarzy et al. [35] in a study involving the interaction of 9-amino-5-alkyl-12(*H*)-chino[3,4-b][1,4]benzothiazine chloride (Salt3) with human serum albumin (HSA). The phenomenon observed by the authors of the paper was correlated with the interaction of Salt3 with a single tryptophanyl residue located in subdomain IIA (Sudlow site I) in the HSA molecule. Based on Owczarzy et al.’s [35] conclusions, it can be speculated that, similar to Salt3, DAPT interacts with HSA in Sudlow site I, which, in turn, may indicate a potentially important role for hydrophobic interaction in the stabilization of the DAPT-HSA complex. The present hypothesis is consistent with the classic model of drug binding to albumin described by Sudlow, Birkett, and Wade [36].

Furthermore, in assessing the interaction between DAPT and HSA, the percentage of HSA fluorescence quenching at 36% (Table 2) requires individual consideration. In the study conducted by Maciążek-Jurczyk et al. [37] involving spectroscopic analysis of the binding ability of phenylbutazone and ketoprofen to serum albumin, the authors of the paper obtained the percentage of fluorescence quenching of HSA by the studied ligands in the range of 60–70%. Based on this, the authors of the paper indicated strong quenching. In contrast to the present work, where the calculated percentage of HSA fluorescence quenching was achieved at 36% for both of the excitation wavelengths, it can be assumed that DAPT quenches HSA fluorescence weakly.

In order to determine the type of HSA fluorescence quenching (static and/or dynamic) in the presence of DAPT at increasing concentrations, the Stern–Volmer equation was applied [38]. Due to the linear course of Stern–Volmer curves at both excitation wavelengths, λ_ex_ 275 nm and λ_ex_ 295 nm (Figure 5), the nature of HSA fluorescence quenching could not be clearly identified, and it was necessary to calculate the Stern–Vomer constant (K_S-V_) and bimolecular quenching constant rate (k_q_). According to Lakowicz’s [39] theory, k_q_ values exceeding 10^10^ indicate static quenching. As shown in Table 4, k_q_ values of the order of magnitude 10^12^ were obtained. In accordance with Lakowicz’s theory [39], these values may indicate that the mechanism of HSA fluorescence quenching in the presence of DAPT has a static nature, involving the formation of a DAPT-HSA complex in the ground state. It is worth noting that the decrease in fluorescence intensity is therefore attributed to the formation of a non-fluorescence complex. Furthermore, the binding of DAPT to HSA molecules may modify the local environment of amino acid residues (fluorophores), reducing the population of those fluorescent forms, which results in lower fluorescence intensity. Additionally, based on a simultaneous analysis of the fluorescence quenching curves (Figure 5) and the assessed mechanism of fluorescence quenching, it can be assumed that a single tryptophan residue (Trp-214) located in subdomain IIA may play a key role in the interaction between the studied ligand and protein.

The association constant (K_a_ [L·mol^−1^]) characterizes the stability of the formed complex. To determine the association constant (K_a_ [L·mol^−1^]) characterizing the stability of the DAPT-HSA complex and to identify the number of binding site classes (n), the Klotz equation was used [40]. Based on Klotz curves (Figure 6, Table 5) and the analysis of the obtained data, it can be observed that the DAPT-HSA complex is characterized by K_a_ values of the order of magnitude 10^4^, and the number of binding site classes oscillated around 1. In study conducted by Vukic et al. [41], where the authors analyzed the binding abilities of natural naphthoquinone derivatives to HSA, they used the Klotz method and obtained significantly higher association constants values (10^7^). Based on this, the authors of the paper assessed the binding affinity for all studied complexes as strong. On the other hand, Mariño-Ocampo et al. [42], analyzing the interaction between Xa factor inhibitors (FXa) and human serum albumin, obtained the association constants of the order of magnitude 10^4^. Thus, they demonstrated that HSA has a moderate affinity for FXa inhibitors. According to literature data, it can be concluded that DAPT forms a complex in one HSA binding site class with moderate affinity.

The thermodynamic parameters of DAPT complex formation with HSA were determined using nano isothermal titration calorimetry (nanoITC). According to obtained values and the nanoITC thermogram (Figure 7), it was determined that the complex formation reaction was exothermic (ΔH < 0) and spontaneous (ΔG < 0), and that the complexation reaction was entropically driven. A positive entropy change (ΔS > 0) results from a significant increase in the number of possible microstates of a water molecule, which is released from a confining space created by the protein tertiary structure into the external environment [43]. Based on this, it can be concluded that DAPT is an example of a hydrophobic, structurally constrained, and entropically driven ligand [44,45]. Additionally, hydrophobic and electrostatic interactions (including ionic bonds) were essential for the stabilization of the studied complex, as evidenced by enthalpy and entropy changes [39]. The hydrophobic effect of ligand–protein interactions can lead to DAPT being transferred from the aqueous solvent into a hydrophobic cavity (Sudlow site I) [46]. Hydrophobic interactions are non-specific, and their selectivity arises primarily from shape complementarity. The formation of a stable DAPT-HSA complex is based on the assumption that the probability of geometrically similar binding pockets occurring at sites other than the primary binding site is very low [47]. Additionally, the nanoITC technique enabled the ascertainment of the stoichiometry of the reaction equal to 5.10 ± 1.23, which suggests that 5 molecules of the studied ligand may probably interact with the protein macromolecule with moderate affinity. These phenomena also confirmed the moderately stable form of the DAPT complex with HSA. These data are an indispensable complement to the spectrofluorescence technique and provide a significant contribution to the information on the interaction of DAPT with HSA. All the data collected are well described by the one-site binding model. This means that approximately one molecule of DAPT is bound per one HSA binding site (Sudlow site I; based on the SFM and nanoITC) and probably a few DAPT particles, which interact superficially with the HSA particle (based on the nanoITC) [48]. So far, no one has studied phenothiazine derivatives using nanoITC. Based on our previous analyses, it can be concluded that the interaction of 9-fluoro-5-alkyl-12(*H*)-quino[3,4-b][1,4]benzothiazine chloride (Salt2) with HSA was described by negative ΔG, ΔH, and ΔS values, while neither the association constant nor the reaction stoichiometry were similar to the results obtained in this paper [49].

NanoDSC is a technique for measuring the thermal transitions of macromolecules. In this study, nanoDSC was used to obtain information about the enthalpy change and to determine the heat capacity of HSA, DAPT-has, and HSA-NPs. In our study, based on molar heat capacity results, HSA as well as DAPT-HSA complex seemed to be stable. In contrast, HSA-NPs were characterized by negative values of the molar heat capacity, probably using the supplied heat for a conformational change (destruction of crosslinking) (Figure 9). Zhang et al. [50] obtained the thermal transition peak (denaturation peak) for native albumin at 53.9 °C. The denaturation temperature for HSA was 60.93 °C, and for DAPT-has, 60.95 °C. In the study by Li et al. [51], the addition of vanillin into BSA nanoparticles shifted the melting peak to a lower temperature, suggesting that chemical reactions occurred during the production of BSA nanoparticles and formed a more stable substance [51]. In the case of native HSA and HSA-DAPT, the denaturation temperatures did not change. The denaturation temperature for HSA-NPs was 65.65 °C, suggesting slightly higher thermal stability of the formed structures compared to the protein from which they were formed. Positive enthalpy values of all of the samples suggested that the transformations were endoenergetic. Attia et al. [52] found that the carvedilol distinctive peak disappeared from the DSC profile of the optimized carvedilol-loaded BSA-based nanoparticles, indicating that the drug was incorporated into the protein structure of the optimized formulation in an amorphous state. As can be seen (Figure 8), DAPT-HSA is more thermally stable than HSA and HSA-NPs. In our studies, we were unable to observe changes for DAPT-HSA-NPs, but it is clear that the modified HSA differed from the native protein. Additionally, the endothermic peak that corresponded to the native BSA ended at 117.35 °C after being moved to 79.56 °C [52]. Due to the limitations of the method, results for DAPT-HSA-NPs at this high temperature could not be obtained.

The circular dichroism (CD) study showed changes in the secondary structure of HSA in the presence of DAPT (Figure 9a), as well as after the nanoparticle preparation process (Figure 9b). A single minimum peak can be observed in Figure 9b, which was also observed in our previous study concerning encapsulated phenothiazine derivative [22]. As shown in Table 6, the nanoparticle preparation process increased the Θ_MRW_ values at both 209 and 220 nm. This may be due to crosslinking of HSA with glutaraldehyde and encapsulation with DAPT. CD is not widely used in terms of nanoparticle study; however, it can provide information on the fate of the protein during nanocarrier preparation [13,15,16,21]. In terms of nanoparticle preparation, the desolvation process had a significant impact on the secondary structure of albumin, which can be seen in Figure 9a,b and was evidenced in our previous studies [13,21]. Organic solvents and the cross-linking process may lead to partial denaturation of albumin, changes to its conformation, and a loss of its natural biological properties. Consequently, this may result in increased immunogenicity of the nanoparticles [17,53]. The effect of changes to the secondary structure of albumin has not yet been investigated.

## 4. Materials and Methods

Human serum albumin (HSA), fraction V (purity minimum 96%) and dimethyl sulfoxide (DMSO) were purchased from Sigma Aldrich Chemie GmbH (Steinheim, Germany). Ethanol was supplied by P.P.H “STANLAB” Sp. z o.o. (Lublin, Poland). Methanol, Lot No. SHBG8324V, was obtained from Sigma Aldrich (St. Louis, MO, USA). Glutaraldehyde was purchased from Warchem (Warsaw, Poland). All chemicals were of analytical grade and were used without further purification. 10*H*-3,6-diazaphenothiazine (DAPT) was synthesized in the Department of Organic Chemistry, Faculty of Pharmaceutical Sciences in Sosnowiec, Medical University of Silesia in Katowice, Poland. The most important properties of DAPT were identified using in silico analysis (SwissADME web tool) [54,55,56].

### 4.1. Nanoparticle Preparation and Characterization

HSA nanoparticles loaded with DAPT (DAPT-HSA-NPs) were prepared using the desolvation method as previously described [14,21]. HSA was dissolved in phosphate buffer, whereas DAPT was prepared in DMSO, maintaining the molar ratio of HSA:DAPT of 1:3. Then, ethanol was added to induce nanoparticle formation, followed by a cross-linking process with an aqueous glutaraldehyde solution. The reaction mixture was stirred for 20 h. After the procedure mentioned, nanoparticle suspension was purified by centrifugation in phosphate buffer (293 K, 13,700× *g*), then was redispersed by vortex and ultrasonicated.

The encapsulation efficiency (EE) was calculated with the use of (Equation (1)), based on UV-Vis measurements performed with a JASCO V-760 spectrophotometer (Hachioji, Tokyo, Japan). Blank HSA nanoparticles (HSA-NPs) were prepared according to the procedure presented but in the absence of the ligand (DAPT). In order to determine the absorption maxima required to calculate the EE, the absorbance of the supernatants obtained after purification of blank nanoparticles (HSA-NPs) was first measured to verify the absorbance values that might potentially affect the measurements of the drug-containing supernatant. The absorbance of both the supernatants for HSA-NPs and for DAPT-HSA-NPs was measured at a wavelength of λ_max_ 250 nm.

(1) $$EE=\frac{total\;ligand\left(mg\right)-free\;ligand\;(mg)}{total\;ligand\;(mg)}\cdot 100\%$$

where total drug (mg) denotes the amount of the 3,6-DAPT added during the preparation process (in mg), whereas free drug (mg) is the amount of the unbound 3,6-DAPT detected in the supernatant (in mg).

The morphology of the nanoparticles was examined using an ultra-high-resolution analytical microscope Apreo 2S (Thermo Fisher Scientific, Waltham, MA, USA). Prior to imaging, the samples were mounted on aluminum stubs and coated with a 5 nm carbon layer using an EM ACE600 sputter coater (Leica Microsystems, Wetzlar, Germany). Microscopic observations were performed under high-vacuum conditions at an accelerating voltage of 5 kV, employing the in-column T3 detector in OptiPlan mode (Thermo Fisher Scientific, Waltham, MA, USA).

### 4.2. In Vitro Ligand Release

The release studies of the nanoparticles were conducted using sample and separate method in 90 mL of phosphate buffer (PBS) (0.05 mol·L^−1^, pH 7.4) at 310 K, under gentle constant stirring. Samples were collected at arbitrary time intervals (0.5, 1, 1.5, 2, 2.5, 3, and 24 h) and filtered through 0.22 μm syringe filters. The filtrates were collected, and the ligand (DAPT) content was measured by the UV-Vis technique with a JASCO V-760 spectrophotometer (Hachioji, Tokyo, Japan) at a wavelength of λ_max_ 250 nm. The sink condition was maintained throughout the entire release studies. The zero-order (Equation (2)) and first-order (Equation (3)) models, as well as the Korsmeyer–Peppas model (Equation (4)), were used to study the in vitro DAPT release kinetics and mechanisms.

The zero-order model was calculated using the following equation (Equation (2)) [29]:

(2) $$C_{r}=C_{0}+K_{0}\cdot t$$

where $C_{r}$ is the concentration of the released ligand [mg·mL^−1^], $C_{0}$ is the initial concentration before the active release in time t ($C_{0}=0\;mg\cdot {mL}^{-1}$), t is the release time [h], and $K_{0}$ is the zero-order constant [h^−1^].

The first-order model was calculated using the following equation (Equation (3)) [29]:

(3) $$\frac{dC}{dt}=-K_{1}\cdot C$$

where C is the concentration of ligand [mg·mL^−1^] and $K_{1}$ is the first-order rate constant [h^−1^].

The Korsmeyer–Peppas model was calculated using the following equation (Equation (4)) [29,30]:

(4) $$\frac{M_{i}}{M_{\infty }}=K_{KP}{\cdot t}^{n}$$

where $\frac{M_{i}}{M_{\infty }}$ is the fractional solute release [mg·mL^−1^], t is the release time [h], $K_{KP}$ is the Korsmeyer–Peppas release constant, and n is the diffusional exponent indicating the transport mechanism.

The simplified Higuchi model was calculated using the following equation (Equation (5)) [29,57,58,59]:

(5) $$Q=K_{H}\cdot \sqrt{t}$$

where Q is the concentration of the released drug [mg·mL^− 1^], t is the release time in minutes, and K_H_ is the release Higuchi constant.

The Hixson-Crowell model was calculated according to the equation (Equation (6)) [29,60]:

(6) $$\sqrt[{3}]{W_{0}}-\sqrt[{3}]{W_{i}}=K_{HC}\cdot t$$

where $\sqrt[{3}]{W_{0}}$ the cube root of the percentage of 3,6-DAPT added, $\sqrt[{3}]{W_{i}}$ the cube root of the percentage of released 3,6-DAPT at time t, $K_{HC}$ is Hixson-Crowell constant, and t is time [h].

### 4.3. Fluorescence Spectra Measurements

Due to the absorption of light at both excitation and emission wavelengths, the fluorescence intensity of albumin, in the absence (HSA) and presence of DAPT (DAPT-HSA) at increasing concentration was corrected (inner filter effect, IFE) using Equation (7) [61]. This equation can be used as long as the increase in the system does not exceed 0.3 near excitation and emission wavelengths:

(7) $$F_{cor}=F_{obs}\cdot e^{\frac{A_{ex}+A_{em}}{2}}$$

where $F_{cor}$ and $F_{obs}$ are corrected and observed fluorescence, respectively, and $A_{ex}$ and $A_{em}$ are the absorbance at the excitation and emission wavelengths, respectively.

The spectral parameter A was used to assess changes in the environment of aromatic amino acid residues and was calculated using Equation (8) [62]:

(8) $$A={\left(\frac{F_{365nm}}{F_{320nm}}\right)}_{cor}$$

where $F_{365nm}$ and $F_{320nm}$ are the fluorescence intensities at λ 365 nm and 320 nm, respectively.

The fluorescence quenching effect (static and/or dynamic) in the DAPT-HSA system was analyzed according to the Stern–Volmer equation (Equation (9)) [33]:

(9) $$\frac{F_{0}}{F}=1+k_{q}\tau_{0}\cdot \left[L\right]=1+K_{S-V}\cdot [L]$$

where F and $F_{0}$ are the fluorescence intensities at the maximum wavelength of albumin in the presence and absence of ligand, respectively, $k_{q}=\frac{K_{S-V}}{\tau_{0}}$ is a bimolecular quenching rate constant [L·mol^−1^·s^−1^], $\tau_{0}$ is the average fluorescence lifetime of albumin without ligand ($\tau_{0\;\left(HSA\right)}$= 6.000 · 10^−9^ s) [23], [L] is the ligand concentration [mol·L^−1^] ($\left[L\right]=\left[L_{b}\right]+[L_{f}]$, where [$L_{b}$] and [$L_{f}$] are the bound and unbound (free) drug concentrations, respectively [mol·L^−1^]), and $K_{S-V}$ is the Stern–Volmer constant [L·mol^−1^] [39].

The association constants ($K_{a}$) in ligand-albumin systems were estimated using the Klotz equation (Equation (10)) [39]:

(10) $$\frac{1}{r}=\frac{1}{n}+\frac{1}{n\cdot K_{a}\cdot [L_{f}]}$$

where r is the number of ligand moles bound to 1 mole of albumin ($r=\frac{L_{b}}{\left[P\right]}$), n is the number of binding sites classes, $K_{a}$ is the association constant [L·mol^−1^], and [$L_{f}$] is the free ligand concentration [mol·L^−1^].

### 4.4. NanoITC Measurements

The calorimetric experiment was performed using nano isothermal titration calorimetry (nanoITC) (TA Instruments, New Castle, DE, USA). Titrations were performed by injecting 50 µL of DAPT solution (1.7 · 10^−3^ mol·L^−1^) into HSA solution (3 · 10^−5^ mol·L^−1^) at 298 K. Before measurements, all solutions (HSA, DAPT) were degassed (degassing time t = 15 min) using Degassing Station (TA Instruments, New Castle, DE, USA). The volume of a single injection was 2.38 µL. As blank (average area), phosphate buffer (pH = 7.4, 5 · 10^−2^ mol·dm^−3^) and DMSO with or without DAPT were used. Each measurement consisted of 20 injections. Solutions were stirred at a rate of 300 rpm throughout the study. The molar ratio of DAPT:HSA ranged from 0.4762:1 to 10.3200:1. All calculations were made based on the software (NanoAnalyze, TA Instruments, USA) provided by the manufacturer.

The Gibbs free energy change ∆G [kcal·mol^−1^] was obtained based on Equation (11) [63]:

(11) ∆G=∆H−T·∆S

where ∆H is the enthalpy change [kcal·mol^−1^], T is the temperature [K], and ΔS is the entropy change [kcal·K·mol^−1^].

### 4.5. NanoDSC Measurements

Thermal properties of the samples (HSA-NPs, DAPT-HSA-NPs, HSA, DAPT-HSA) were analyzed using a nano differential scanning calorimetry (NanoDSC) (TA Instruments, New Castle, DE, USA). Solutions of HSA-NPs and DAPT-HSA-NPs (at 3 · 10^−5^ mol·L^−1^ concentration), as well as HSA and DAPT-HSA (at 3.5 mg·mL^−1^ concentration), were added to a 96-well plate and placed in an autosampler. Samples were heated from 313 K to 403 K at the heating rate of 1 °C·min^−1^ at 5 bar. All samples were degassed before analysis (degassing time t = 15 min) using Degassing Station (TA Instruments, New Castle, DE, USA). The thermograms were prepared for analysis by subtracting the buffer scan from the sample and converting to molar heat capacity values. The denaturation temperature $T_{D}$ [℃] and enthalpy change ΔH [kcal·mol^−1^] were determined [64,65] as the peak temperature and the area under the heat flow curve, respectively, and the enthalpy was normalized to J·g^−1^, using the software (NanoAnalyze, TA Instruments, USA) provided by the manufacturer.

### 4.6. Circular Dichroism Measurements

Circular dichroism (CD) spectra of native HSA, HSA in the presence of DAPT (DAPT-HSA), HSA nanoparticles (HSA-NPs), and HSA nanoparticles with encapsulated DAPT (DAPT-HSA-NPs) were recorded using a Jasco J-1500 spectropolarimeter (Hachioji, Tokyo, Japan). The spectrum of DAPT was recorded as a reference. The measurements were conducted at 293 K, in quartz cuvettes with an optical path of 1 mm. The spectra were recorded in the wavelength range from 200 to 250 nm (secondary structure image). The accuracy of the wavelength measurement was ±0.1 nm, the wavelength repeatability was ±0.05 nm, and the scanning speed was 20 nm·min^−1^. The percentage content (%) of HSA secondary structure elements was determined based on the ContinLL method and reference set 4 on the Dichroweb [66]. The mean residue ellipticity ${\left[\theta \right]}_{MRW}$ was obtained by Equation (12) [38,66]:

(12) $${\left[\Theta \right]}_{MRW}=\frac{MRW\cdot \Theta }{10\cdot l\cdot c}$$

where Θ is the observed ellipticity for a given wavelength [deg], l is the pathlength [cm], c is the albumin concentration [g·cm^−3^], and MRW is the mean residue weight (${MRW}_{HSA}$ = 114.7 Da) [38,66].

### 4.7. Statistical Analysis

At least three repetitions were conducted for each sample. Figure 1 was prepared using ChemSketch 12.1.0.31258 software. Utilizing the Spectra Manager Version 2.13.00 2002–2015 software, spectroscopic spectra were examined. Size analysis of the nanoparticles was performed in ImageJ 1.53k software. OriginPro version 8.5 SR1 software was used to visualize the study’s results as a mean ± relative standard deviation (SD).

## 5. Conclusions

This research is an attempt to encapsulate the new phenothiazine derivative structure of 10*H*-3,6-diazaphenothiazine (DAPT) in human serum albumin (HSA) nanoparticles and show the fate of the released drug in vitro, from the moment the substance is released, through its interaction with albumin. The obtained drug delivery system has been characterized by diameter of 114 nm (HSA-NPs), and their diameter increased to 120 nm following the encapsulation of DAPT. In order to ascertain the release kinetics mechanism, which is essential for the particles’ medical applications, mathematical methods were implemented. In vitro, the activity of nanocarriers can be evaluated by calculating release kinetics and plotting the release of DAPT from nanoparticles. According to the results obtained, DAPT-HSA-NPs release according to the zero-order kinetics, and the release mechanism estimated from the Korsmeyer–Peppas model was similar to that described in the literature for other albumin nanoparticles (Super Case II transport). Spectrofluorescence analysis allowed the conclusion that DAPT forms a static, non-fluorescent complex, most likely at Sudlow site I (subdomain IIA), with moderate affinity and calorimetric studies suggesting that one HSA molecule can react with at least 4 DAPT molecules. The secondary structure of HSA was also altered under the influence of DAPT, but we do not know whether changes at the molecular level affect the entire system in vivo.

The above results indicate the potential applicability of DAPT-HSA-NPs in medicine. Due to the insufficient number of studies on DAPT-containing nanoparticles, despite of promising results, further studies on albumin nanoparticles with this phenothiazine derivative are necessary to further investigate the therapeutic potential of the obtained structures. This study may also provide a basis for further preclinical and clinical studies.

## Figures and Tables

**Table 1:** Mathematical models of release kinetics of DAPT from DAPT-HSA-NPs.

**zero-order model**	R^2^ ± SD *	0.999 ± 0.004
	K_0_ ± SD * [1·h^−1^]	6.33 ± 1.71
**first-order model**	R^2^ ± SD *	0.72 ± 0.06
	K_1_ ± SD * [1·h^−1^]	0.31 ± 0.01
**Korsmeyer–Peppas model**	R^2^ ± SD *	0.99 ± 7.07 · 10^−5^
	K_KP_ ± SD *	1.1 ± 0.17
	n ± SD *	0.95 ± 0.02
**Higuchi model**	R^2^ ± SD *	0.98 ± 0.02
	K_H_ ± SD *	0.67 ± 0.03
**Hixson–Crowell model**	R^2^ ± SD *	0.99 ± 1.70 · 10^−3^
	K_HC_ ± SD *	−0.20 ± 0.18

* SD—standard deviation.

**Table 2:** Percentage [%] of HSA fluorescence quenching at the highest DAPT concentration (λ_ex_ 275 nm and λ_ex_ 295 nm).

Molar Ratio Ligand:Albumin	Percentage of Fluorescence Quenching [%] ± SD *	
	λ_ex_ 275 nm	λ_ex_ 295 nm
**10:1**	35.81 ± 2.43	36.38 ± 0.59

* SD—standard deviation.

**Table 3:** Spectral parameter A of HSA at 2 · 10^−6^ mol·L^−1^ concentration in the absence and presence of DAPT (λ_ex_ 275 nm and λ_ex_ 295 nm).

Albumin	DAPT [mol·L^−1^]	275 nm		295 nm	
**HSA**		λ_max_ ± SD * **[nm]**	A ± SD *	λ_max_ ± SD * **[nm]**	A ± SD *
	0	335.00 ± 0.00	0.81 ± 0.01	340.00 ± 0.00	1.19 ± 0.01
	2.0 · 10^−5^	333.50 ± 0.71	0.80 ± 0.01	341.30 ± 0.57	1.29 ± 0.01

* SD—standard deviation.

**Table 4:** Stern–Volmer (K_S-V_) and bimolecular quenching rate (k_q_) constants of the DAPT-HSA system (λ_ex_ 275 nm and λ_ex_ 295 nm).

System	λ_ex_ 275 nm		λ_ex_ 295 nm	
**DAPT-HSA**	(K_s-v_ ± SD *) · **10^4^** **[** **L** · **mol^−1^]**	(k_q_ ± SD *) · **10^12^** **[L·mol^−1^·s^−1^]**	(K_s-v_ ± SD *) · **10^4^** **[L·mol^−1^]**	(k_q_ ± SD *) · **10^12^** **[L·mol^−1^·s^−1^]**
	2.59 ± 0.05	4.32 ± 0.09	2.76 ± 0.05	4.60 ± 0.09

* SD—standard deviation.

**Table 5:** The binding parameters for the DAPT-HSA system at λ_ex_ 275 nm and λ_ex_ 295 nm excitation wavelengths.

System	λ_ex_ 275 nm		λ_ex_ 295 nm	
	(K_a_ ± SD *) · 10^4^ [L·mol^−1^]	n ± SD *	(K_a_ ± SD *) · 10^4^ [L·mol^−1^]	n ± SD *
**DAPT-HSA**	7.85 ± 0.53	0.97 ± 0.06	5.26 ± 0.01	0.95 ± 0.01

* SD—standard deviation.

**Table 6:** The mean residue ellipticity ([Θ]MRW) of albumin secondary structure elements, both native and modified, respectively, in the absence (HSA, HSA-NPs) and presence of ligand (DAPT-HSA, DAPT-HSA-NPs).

System	Θ_MRW_ at 209 nm ± SD * [deg·cm^2^·dmol^−1^]	Θ_MRW_ at 220 nm ± SD * [deg·cm^2^·dmol^−1^]
**HSA**	−17,827.40 ± 355.89	−16,516.92 ± 286.79
**DAPT-HSA**	−1355.12 ± 360.58	−15,204.21 ± 48.74
**HSA-NPs**	−942.92 ± 140.19	−6472.16 ± 48.55
**DAPT-HSA-NPs**	−579.84 ± 138.47	−5674.54 ± 92.23

* SD—standard deviation.

## Data Availability

The original contributions presented in this study are included in the article and Supplementary Materials. Further inquiries can be directed to the corresponding author.

## Supplementary Materials

The following supporting information can be downloaded at: https://www.mdpi.com/article/10.3390/molecules31142541/s1, Figure S1: The most representative steady state fluorescence spectra of DAPT-HSA studied and the corresponding shift (a) λ_ex_ 275 nm, (b) λ_ex_ 295 nm.

## Abbreviations

The following abbreviations are used in this manuscript:

HSA	Human serum albumin
DAPT	10*H*-3,6-diazaphenothiazine
DAPT-HSA-NPs	Human serum albumin nanoparticles with 10*H*-3,6-diazaphenothiazine
DAPT-HSA	Interaction of 10*H*-3,6-diazaphenothiazine with human serum albumin
HSA-NPs	Human serum albumin nanopartricles
nanoITC	Nano isothermal titration calorimetry
DSC	Differential scanning calorimetry
TD	Denaturation temperature
CD	Circular dichroism
[Θ]MRW	The mean residue ellipticity
