# Supplementary material for: Human Serum Albumin Nanoparticles as 3,6-Diazaphenothiazine Delivery System: Preparation and Interaction Studies
Source: Molecules. 2026 Jul 22;31(14):2541. doi: 10.3390/molecules31142541 (PMC13415044; doi:10.3390/molecules31142541)
Supplement: Supplementary file 1 [file molecules-31-02541-s001.zip › molecules-4358016-supplementary.pdf]

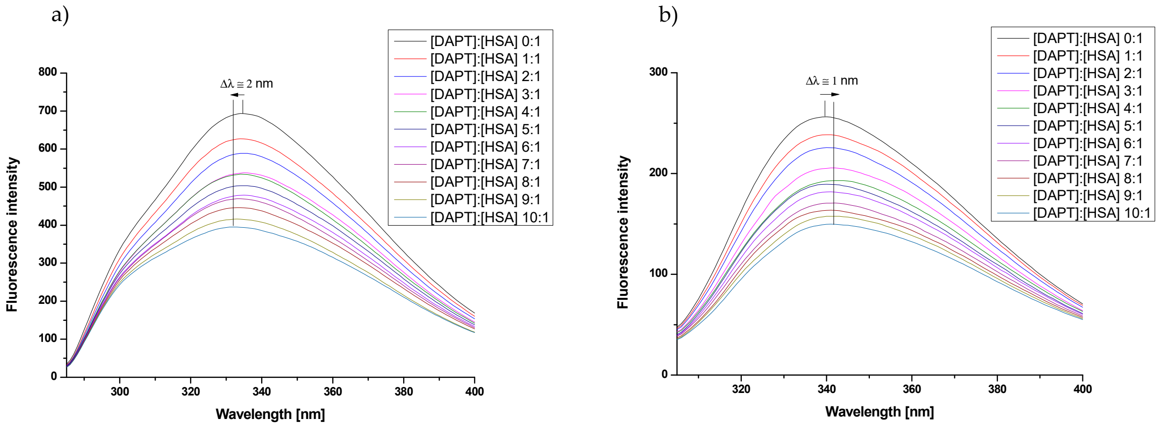

Figure S1. The most representative steady state fluorescence spectra of DAPT-HSA studied and the corresponding shift (a) λ_{ex} 275 nm, (b) λ_{ex} 295 nm
